# Biosynthesis of Silver Nanoparticles by *Aspergillus terreus*: Characterization, Optimization, and Biological Activities

**DOI:** 10.3389/fbioe.2021.633468

**Published:** 2021-04-15

**Authors:** Walid A. Lotfy, Basma M. Alkersh, Soraya A. Sabry, Hanan A. Ghozlan

**Affiliations:** ^1^Department of Microbiology, Faculty of Dentistry, Pharos University in Alexandria, Alexandria, Egypt; ^2^Marine Environment Division, National Institute of Oceanography and Fisheries, Alexandria, Egypt; ^3^Department of Botany and Microbiology, Faculty of Science, Alexandria University, Alexandria, Egypt

**Keywords:** *Aspergillus terreus*, silver nanoparticles, fractional factorial design, cytotoxicity, antimicrobial

## Abstract

In this study, mycelial filtrate of *Aspergillus terreus* BA6 was used to reduce AgNO_3_ to form silver nanoparticles (AgNPs). The effect of seven independent variables on the diameter of AgNPs was studied by applying design of experiments (DOE). At optimal conditions, the diameter of AgNPs was reduced by approximately 26.7% compared to the basal culture condition and AgNO_3_ concentration was found to be the most significant factor affecting the diameter of AgNPs. *A. terreus* nano-Ag was characterized using UV-visible spectroscopy, transmission electron microscopy, energy dispersive X-ray (EDX), X-ray diffraction (XRD), Fourier transform infrared spectroscopy (FTIR), and Zeta potential. The maximum UV absorption was obtained at 420 nm and the microscopic results showed particles with narrow size distribution ranging from 7 to 23 nm. XRD pattern of AgNPs revealed four diffraction peaks of metallic silver and the EDX spectrum showed a strong signal attributed to Ag nano-crystals. AgNPs mycofabricated by *A. terreus* showed potent minimum inhibitory concentration (MIC) and broad minimum bactericidal/fungicidal concentration (MBC/MFC) against 12 reference microorganisms. The MIC and MBC/MFC values of AgNPs were 0.312 to 1.25 μg/ml and 0.625 to 10 μg/ml, respectively. Nevertheless, AgNPs did not demonstrate any antagonistic activity against Coxsackie B virus. The *in vitro* cytotoxicity of the mycosynthesized AgNPs showed significant antitumor activity against adenocarcinoma epithelial cells from human breast cancer (Mcf-7) cell line with an inhibitory concentration (IC_50_) of 87.5 μg/ml.

## Introduction

Silver nanoparticles (AgNPs) are used in various fields such as renewable energy, water treatment, sensors, electronics, the textile industry, and medical applications (Abdelghany et al., 2017). In the last decade, AgNPs have been paid special attention due to the reduction in antibiotics efficiency and development against highly resistant microbes (Ebrahiminezhad et al., 2017; Lotfy et al., 2018b).

Compared to bacteria, fungi produce much higher amounts of proteins, leading to a significant increase in AgNPs productivity (Sastry et al., 2003). Extracellular synthesis of AgNPs has been investigated using many molds such as *Rhizopus arrhizus*, *Trichoderma gamsii*, *Aspergillus niger*, *Penicillium aurantiogriseum*, and *Verticillium chlamydosporium* (Mekawey and Helmy, 2017; Ottoni et al., 2017). However, there are only few reports on the mycosynthesis of AgNPs by *Aspergillus terreus* (Li et al., 2012; Raliya and Tarafdar, 2012; Abd et al., 2013; Abdel-Hadi et al., 2014; Sayed et al., 2015).

Optimization of the factors affecting the features of AgNPs is crucial for an up-scalable and controllable synthesis process. These factors include; biomass and substrate concentration, exposure time, pH, temperature, mixing speed, metal ion concentration, inoculum size, ratio of extract to silver nitrate, and composition of fermentation medium (Krishnaraj et al., 2012; Korbekandi et al., 2013; Saxena et al., 2016). Furthermore, AgNPs have extremely different properties compared to their bulky sized counterparts, owing to the boosted surface to volume ratio in the former (Srikar et al., 2016). Since surface atoms are more reactive than atoms on the center, nanomaterials are more reactive than their larger size materials. Therefore, reducing the size of nanomaterials is expected to boost their reactivity and enhance their properties (Prasad et al., 2016). To the best of our knowledge, there has been no study investigating the optimization of nano-silver synthesis by *A. terreus* for a reliable biogenic industrial application.

Design of experiments (DOE) such as fractional factorial designs are sets of statistical and mathematical tools used for analysis and modeling of the processes where the response is influenced by several variables (Lotfy et al., 2018a). DOE are generally used to identify the most significant parameters in the bioprocess and to predict the optimal conditions (El-Helow et al., 2019; Lotfy et al., 2019). DOE are more effective, reliable and consume less materials, time, and effort to determine the near optimum settings for each variable. Recently, these designs have been successfully employed in many bioprocess optimizations (Lotfy et al., 2017, 2018a; Ghanem et al., 2020).

Potential treatment for drug resistant microorganisms is a rapidly rising threat to public health as our resources of effective antibiotics decline (Lotfy et al., 2018b). Therefore, the development of new antimicrobial agents is strongly demanded. AgNPs are significant nanomaterials with excellent antimicrobial activity that ensues from cell wall and membrane disruption, damage of nucleic acid, and generation of reactive oxygen species (ROS) (Rai et al., 2016; Fanoro and Oluwafemi, 2020).

Several studies have reported that AgNPs demonstrate potent antiviral action against several pathogenic viruses such as herpes simplex virus, influenza virus, respiratory syncytial virus, human immunodeficiency virus, and hepatitis B virus (Galdiero et al., 2011). On the other hand, there is a lack of investigations concerning the antiviral activity of AgNPs and their mechanism of action against various viruses (Huy et al., 2017). In the current study, we aimed to examine the antiviral activity of *A. terreus* nano-Ag against Coxsackie B virus.

Globally, cancer as a life-threatening disease accounts for high mortality rates (Al Ansari et al., 2020). The physical and structural characteristics of AgNPs granted them the facility to penetrate and destroy cancer cells. AgNPs express their cytotoxic activity via oxidative stress and through production of ROS that causes DNA damage and mitochondrial related apoptosis and necrosis (Datkhile et al., 2017).

The main objectives of the current study are: (1) to select and identify a competent fungal isolate capable of producing AgNPs, (2) to reduce the size of AgNPs through optimization of the synthesis parameters, and (3) to test the antimicrobial, antiviral, and antitumor activities of the produced AgNPs.

## Materials and Methods

### Microorganisms

*Aspergillus terreus* strain BA6 was used for the synthesis of AgNPs. The strain was isolated from soil and identified by DNA sequencing. The sequence was deposited in NCBI GenBank (Accession Number MG725681^1^). Two copies of the fungus were deposited in Assiut University Mycological Centre, Egypt (Accession No. AUMC14437) and in the Egyptian Microbial Culture Collection Network, Egypt (Accession No.  EMCCN2784). *Bacillus subtilis* (ATCC 6633, Gram positive), *Escherichia coli* (ATCC 8739, Gram negative), *Pseudomonas aeruginosa* (ATCC 9027, Gram negative), *Salmonella typhimurium* (ATCC 14028, Gram negative), *Staphylococcus aureus* (ATCC 6538, Gram positive), *Staphylococcus epidermidis* (ATCC 12228, Gram positive), *Streptococcus faecalis* (ATCC 10541, Gram positive), Methicillin resistant *Staphylococcus aureus* (MRSA) (ATCC 43300, Gram positive), *Listeria monocytogenes* (ATCC 19111, Gram positive), *Aeromonas hydrophila* (ATCC 35654, Gram negative), *Candida albicans* (ATCC 10231), and *Aspergillus niger* (ATCC 16404) were used in the determination of minimal inhibitory and bactericidal/fungicidal concentrations (MIC and MBC/MFC) of AgNPs.

### Cell Lines

Mcf-7 cell line (ATCC No. HTB-22), an adenocarcinoma epithelial cells from human breast cancer, and Vero cell line (ATCC No. CCL-81), an epithelial kidney cells from African green monkey, were used to test the cytotoxicity and antiviral activity of AgNPs.

### Virus

Coxsackie B virus (COXB4) was obtained from VACSERA^®^ (Cairo, Egypt).

### Chemicals and Media

All chemicals used in the present work were of analytical grade. Silver nitrate (AgNO_3_) is a product of Gamma Chemicals, United States. Dextrose, peptone, and sodium chloride are products of El-Nasr Pharmaceutical and Chemicals Company, Egypt. Agar-Agar powder (Research-Lab Fine Industries Company, India): seventeen grams of agar were added to broth media for the preparation of solid media. All media were prepared and sterilized according to the manufacturer instructions. The following culture media were used: Sabouraud dextrose broth (SDB) medium (Condalab, Spain) is of the following composition (g/l): Dextrose, 20; mixture of peptic digest of animal tissue and pancreatic digest of casein (1:1), 10. Muller Hinton broth (MHB) medium (Oxoid) composed of (g/l): Beef, dehydrated infusion form, 300; casein hydrolysate, 17.5; starch, 1.5.

### Fungal Strain Isolation and Identification

*Aspergillus terreus* BA6 was isolated from soil according to Kumar et al. (2015) and was tested for the ability of its extract to reduce AgNO_3_ into AgNPs. The isolate was identified molecularly by DNA extraction followed by PCR to amplify the following segments of rRNA gene; internal transcribed spacer 1 (ITS1) sequence (partial), 5.8S (complete sequence) and internal transcribed spacer 2 (ITS2) sequence (partial). ITS1 forward primer (TCCGTAGGTGAACCTGCGG) and ITS2 primer (TCCTCCGCTTATTGATATGC) were used. The purified PCR product was sequenced using 3730XL DNA Analyzer (Applied Biosystems^TM^). Blast program was used to assess the DNA similarities via multiple sequence alignment. The phylogenetic tree was displayed using MEGA 6 software.

### Biosynthesis of AgNPs by *A. terreus* BA6

A flask containing 100 ml sterile SDB was inoculated with 1 ml of spore suspension (10^6^ spores/ml) and incubated for 5 days at 25°C under shaking at 200 rpm (Gadea et al., 2013). The culture was filtered and the biomass was washed three times with sterile deionized water (Jaidev and Narasimha, 2010). Approximately 20 g wet weight of fungal mycelia were transferred to 100 ml of sterile deionized water and incubated for 2 days at 25°C, 200 rpm. Mycelial pellets were removed via filtration and 200 μl of 0.5M AgNO_3_ solution were added to 100 ml fungal filtrate to obtain a final concentration of 1 mM AgNO_3_. The mixture was incubated at 25°C, 200 rpm for 2 days in the dark (Hemath et al., 2010). After incubation, the mixture was checked for brown color which indicated the formation of AgNPs. The particles were harvested by ultracentrifugation at 4°C, 20,000 rpm for 15 min and freeze dried using bench top lyophilizer (VirTis Freeze Dryer sentry 2.0) (Metuku et al., 2014). A schematic diagram of AgNPs synthesis mechanism is shown in Supplementary Figure 1.

### Optimization of AgNPs Diameter

To evaluate the relative importance of various variables affecting the size of the produced AgNPs; a two-level fractional factorial design, the Plackett-Burman experimental design (Plackett and Burman, 1946), was applied. Seven independent variables were studied; medium pH, ratio of mycelial filtrate to AgNO_3_ (V/V), reaction pH, inoculum size (spores/ml), dextrose (g/l), peptone (g/l), and AgNO_3_ concentration (mM). For each variable, high (+) and low (−) levels were tested (Supplementary Table 1). All trials were carried out in triplicates and incubated for 5 days at 25°C under shaking at 200 rpm. The response, average diameter of AgNPs, was measured using Nano and Zetasizer (Malvern, United Kingdom). The main effect, statistical *t*-values and *p*-values for each variable were calculated using Statistica 10 software. For the purpose of model validation of the results revealed by the Plackett-Burman experimental design, a confirmatory experiment was designed and the basal culture condition of the Plackett-Burman experiment was used as a control. In addition, a reverse state to the optimized culture condition was applied for comparison.

### UV-Visible Spectroscopy

UV-visible spectrum of the mycosynthesized AgNPs was checked between 300 and 600 nm using Helios Alpha, 9423 UVA 1002E spectrophotometer to ensure the presence of specific Surface Plasmon Resonance (SPR) peak of AgNPs. The crude fungal filtrate with freshly added silver nitrate was used as a blank.

### High Resolution Transmission Electron Microscopy (HRTEM)

Silver nanoparticles were imaged using field emission transmission electron microscope (JEOL) which operates at 190 keV. The samples were prepared according to Ndikau et al. (2017) and the diameters of AgNPs were measured using the software of the microscope.

### X-ray Diffraction (XRD)

Diffraction pattern, size, crystallinity, and lattice parameter of AgNPs were determined via X-ray diffractometer (Shimadzu Xlab 6100) operated at 40 kV and 30 mA. The spectrum was recorded by CuKa radiation with wavelength of 1.54606A in the 2θ range of 5°–80° with a step of 0.02 degrees (Bykkam et al., 2015). A glass slide was drop-coated with AgNPs solution and air dried, making a heavy film of AgNPs on the slide (AbdelRahim et al., 2017). Average size of AgNPs was estimated using Debye-Scherrer formula (Patterson, 1939) as following:

D=0.9⁢λ/β⁢cos⁡θ

Where “D” is the particles diameter, “λ” is the wavelength of X-ray (nm), “β” is the full width at half maximum (FWHM) in radians, and “θ” is the diffraction angle. Bragg’s law was used to calculate the value of *d*-spacing (*d*) which is the distance between planes of atoms inside the crystal (Bykkam et al., 2015).

2⁢d⁢sin⁡θ=n⁢λ

d=λ/2sinθ(n=1)

### Energy Dispersive X-ray (EDX) Spectroscopy

Elemental analysis was performed using INCAPENTA-FET attached with scanning electron microscope JEOL JSM-5300. A drop of AgNPs suspension was placed onto a carbon-coated copper grid, dried by air, and used for analysis (Ottoni et al., 2017); afterward an X-ray was generated from the whole scan area. Y-axis represents count per second (CPS) which is the number of X-rays the detector received and processed; whereas, the X-axis represents the energy level of the striking beam.

### Size Distribution and Zeta Potential (ξ)

Zeta potential and the preliminary AgNPs size distribution were determined by Dynamic Light Scattering (DLS) using Malvern Zetasizer Nano ZS (Malvern Instruments, Malvern, United Kingdom) analyzer at room temperature with 90° detection angle (Xue et al., 2016).

### Fourier Transform Infrared Spectroscopy (FTIR)

Fourier transform infrared spectroscopy measurement aimed to define the functional groups of biological molecules responsible for the synthesis and capping of AgNPs (Heera and Shanmugam, 2015). Potassium bromide was added to the dried AgNPs powder (100:1) and the FTIR spectrum was recorded using FTIR Spectrophotometer (Perkin Elmer) in the range of 450–4,000 cm^–1^ (Roy et al., 2013).

### Determination of the MIC, MBC, and MFC of AgNPs

MIC, MBC, and MFC of AgNPs against 12 ATCC standard microorganisms were determined by applying broth microdilution protocol, as recommended by the guidelines of Clinical Laboratory Standards Institute (CLSI), using 96 well microtiter plates (Clinical and Laboratory Standards Institute [CLSI], 2012). Two-fold serial dilutions of known concentrations of AgNPs ranging from 0.019 to 10 μg/ml were prepared using MHB. To each well, 25 μl inoculum of pathogens (5 × 10^5^ cfu/ml) and 25 μl of MHB containing AgNPs or standard antibiotics or mycelial free extract were added. The antimicrobial activity of sterile crude mycelial free extract was tested against each pathogen as a negative control. In addition, three wells contained only MHB were utilized to test the sterility of the assay media. Inoculated MHB media with standard antibiotics were used as positive control. After inoculation with pathogens, bacterial cultures were incubated at 37°C for 24 h while fungal cultures were incubated at 25°C for 72 h. After incubation, the lowest concentration of AgNPs which completely inhibited the visible growth of microbes was recorded as the MIC. A loopful of inoculum was taken from each well showing no visual growth after incubation; spotted onto MHA and incubated at 37°C for 72 h or spotted onto SDA and incubated at 25°C for 5 days. The previous step was necessary to validate the MIC assay and to determine the MBC and the MFC values. The lowest concentration of AgNPs which completely killed and prevented the growth of microbes on agar media was recorded as the MBC or MFC.

### Assessment of the Antiviral Activity of AgNPs Against Coxsackie B Virus

MTT (3-(4, 5-Dimethyl thiazol-2yl)-2, 5-diphenyl tetrazolium bromide) assay was used to assess AgNPs ability to inhibit the *in vitro* infectivity of coxsackie B virus on Vero cell line (Gaikwad et al., 2013). The MTT reduction assay was used to determine the maximum non-toxic concentration (MNTC) of AgNPs on Vero cell line, then, equal volumes (1:1, V/V) of AgNPs (MNTC) and Coxsackie B viral suspension were mixed and incubated for 1 h. A volume of 100 μl of that mixture was added to the wells containing cells. Wells that receive 100 μl of viral suspension without AgNPs were treated as control. Next, aliquots of 200 μl of MTT solution were added to each well and the plate was shaken for 5 min at 150 rpm and incubated at 37°C for 5 h in a humidified chamber containing 5% CO_2_ to allow the MTT to be metabolized. Then, the spent media was decanted, formazan in each well was dissolved in 200 μl dimethyl sulfoxide (DMSO), and the plate was shaken for 5 min at 150 rpm. The optical density (OD) of each well was recorded at 560 nm using a microtiter plate reader (Hu et al., 2014) and directly correlated to cells viability. The viability of the cells inoculated with viral suspension was compared to the viability of the cells infected with Coxsackie B virus and treated with AgNPs.

### Assessment of the Cytotoxic Activity of AgNPs

MTT colorimetric assay was used to assess the cytotoxicity of AgNPs mycosynthesized by *A. terreus* BA6 against Mcf-7. This *in vitro* colorimetric assay depends on the split of MMT by NAD-dependent dehydrogenase of viable cells, resulting in formazan. The purple color of formazan is proportional to the number of viable cells directly and to the cytotoxicity degree inversely (El-Naggar et al., 2017; Lotfy et al., 2018b). Briefly, after culturing a complete monolayer sheet of Mcf-7 cells, growth medium was decanted and fresh RPMI media with 2% serum containing double fold dilutions of AgNPs, ranging from 700 to 5.46 μg/ml, were added and incubated at 37°C for 24 h. Aliquots of 20 μl MTT solution were added to each single well after decantation of the spent media and the plate was incubated at 37°C for 4 h in a humidified chamber containing 5% CO_2_. Thenceforth, MTT solution was decanted from the plate and replaced with 200 μl of DMSO in each well and mixed thoroughly. The OD was read at 560 nm with microplate spectrophotometer. Cells not exposed to AgNPs were used as control (Venkatesan et al., 2014). The absorbance was directly representing the quantity of living cells and the inhibition rate of cells was calculated using the following formula (Hu et al., 2014):

$$Inhibitionrate\left(\%\right)=\left(\frac{O\,D_{c\,o\,n\,t\,r\,o\,l\,g\,r\,o\,u\,p}-O\,D_{t\,e\,s\,t\,g\,r\,o\,u\,p}}{O\,D_{c\,o\,n\,t\,r\,o\,l\,g\,r\,o\,u\,p}}\right)\times 100$$

The resulting data was used to estimate the half maximal inhibitory concentration, IC_50_ (Sherif et al., 2015). The degree of selectivity of AgNPs toward Mcf-7 cells was expressed by the selectivity index (SI) value according to the following equation:

$$\mathrm{SI}={\mathrm{IC}}_{50}\,\mathrm{Vero}\,\mathrm{cells}/{\mathrm{IC}}_{50}\,\mathrm{Mcf}\,\text{-}\,7\,\mathrm{cells}.$$

SI value greater than 2.0 indicates its selective toxicity (Artun et al., 2016).

### Statistical Analysis

Experiments were performed in triplicates, the standard deviation and the mean were calculated, and *p*-value of 0.001 was considered significant. For optimization purposes, the Plackett-Burman design was adopted which is a form of multivariate non-linear regression.

## Results

### Identification of the Isolated Fungus

BA6 isolate was identified molecularly via PCR and the size of the amplified PCR product was 934 bps. The Sanger’s dideoxy nucleotide sequencing of the amplified product resulted in 543 bp nucleotide sequence. The BLASTn analyses and sequence alignment (pairwise and multiple) revealed 99–100% identity with the sequences of several *A. terreus* strains and the isolate was designated as *A. terreus* strain BA6. The sequence has been deposited in NCBI GenBank under accession number MG725681 and the phylogenetic tree is shown in Supplementary Figure 2.

### Detection of AgNPs Formation Using UV-Visible Spectroscopy

**FIGURE 1:**
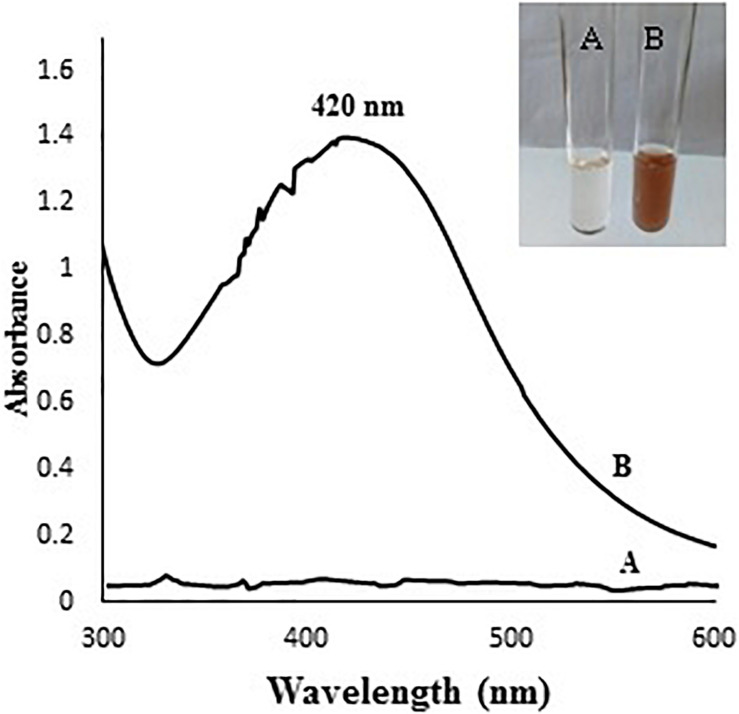
**(A)** colorless mycelial filtrate, **(B)** brown color due to AgNPs formation. The chart shows UV-Visible absorption spectrum of *A. terreus* BA6 AgNPs after 72 h.

The color of mycelial filtrate turned from yellow to dark brown after incubation with AgNO_3_ for 2 days (Figure 1); the color change reflects a primary indication for AgNPs formation (Questera et al., 2013; Elgorban et al., 2016). The specific absorption peak of AgNPs occurs in the visible range of 380–450 nm depending on their size, shape and particle interaction with the medium such as agglomeration (Elgorban et al., 2016). Therefore, UV-visible spectrometry was used to confirm the presence of AgNPs in the range of 300–600 nm. As shown in Figure 1, the specific SPR peak of AgNPs was found to be centered at 420 nm in the spectrum confirming the presence of AgNPs.

### Screening for the Significant Variables Affecting the Size of AgNPs

The Placket-Burman design was applied as described in the Materials and Methods section and the average diameter of AgNPs was determined by DLS as presented in Supplementary Table 1. In trials number 1, 4, and 7 no formation of AgNPs was observed. ANOVA test was established and the *p*-value for each observed response was determined to analyze the relationship between the variables at 99% or higher confidence level (Supplementary Table 2). It can be observed from the degree of significance that the linear term of variables was significant at 1% level. The values of R^2^ (0.99989), adjusted R^2^ (0.99964), and standard error (0.003786) indicate that the model as fitted explains 99.9% of the variability in the biosynthesis of AgNPs. The main effect of each studied variable shows that the seven variables have a significant effect on the size of AgNPs. It is obvious that the most significant factors affecting the size of AgNPs were AgNO_3_ (mM) followed by medium pH, peptone concentration (g/l), filtrate to AgNO_3_ ratio, reaction pH, dextrose (g/l), and finally the inoculum size (spores/ml). AgNO_3_ concentration, medium pH, peptone concentration, reaction pH, and inoculum size positively affected the diameter of AgNPs. On the other hand, filtrate to silver nitrate ratio and dextrose concentration negatively affected AgNPs diameter. Using Statistica 10 software, the following polynomial function was fitted to the experimental response results:

$$Z=458.11+253.40\,X_{1}-111.22\,X_{2}+91.22\,X_{3}+48.22\,X_{4}-66\,X_{5}+208.17\,X_{6}+417.82\,X_{7}$$

**FIGURE 2:**
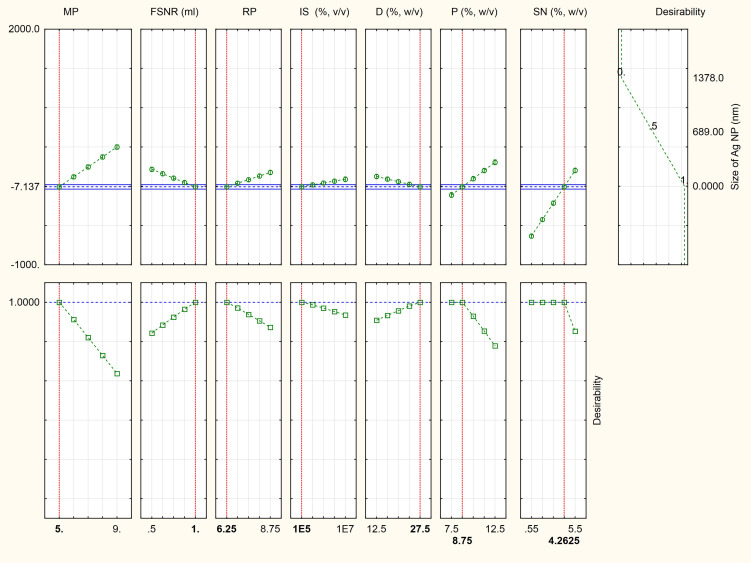
Desirability charts of variables for maximum response. MP, medium pH; FSNR, filtrate to silver nitrate ratio; RP, reaction pH; IS, inoculum size (spores/ml); D, dextrose (g/l); P, peptone (g/l); and SN, silver nitrate (mM).

Where *Z* is the response (size of AgNPs, nm), *X*_1_, *X*_2_, *X*_3_, *X*_4_, *X*_5_, *X*_6_, and *X*_7_ are, respectively, the medium pH, filtrate to AgNO_3_ ratio, reaction pH, inoculum size (spores/ml), dextrose (g/l), peptone (g/l), and silver nitrate (mM). As shown in the desirability chart (Figure 2), the model revealed a near optimum response at the following levels: medium pH, 5; filtrate to AgNO_3_ ratio, 1; reaction pH, 6.25; inoculum size, 10^5^ (spores/ml); dextrose, 27.5 (g/l); peptone, 8.75 (g/l); and AgNO_3_, 4.26 mM.

**FIGURE 3:**
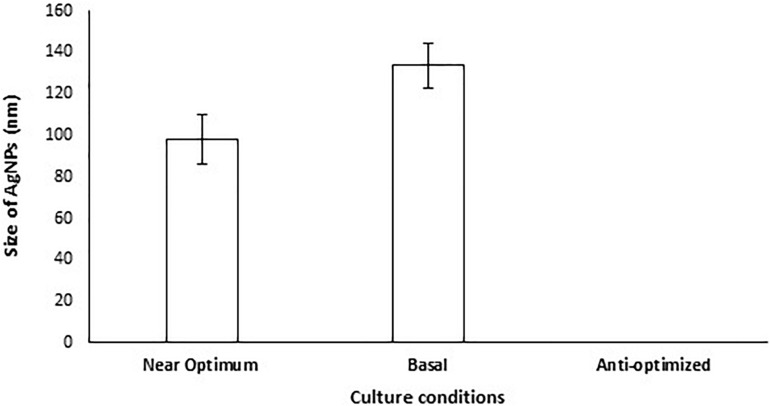
Size of AgNPs mycosynthesized by *A. terreus* BA6 in basal, anti-optimized and optimized culture conditions.

A validation experiment was performed under the parameter settings presented in Supplementary Table 3. The results revealed that no AgNPs were formed when *A. terreus* BA6 filtrate was exposed to AgNO_3_ under anti-optimized conditions ensuring the model accuracy (Figure 3). On the other hand, near optimum conditions resulted in 26.7% smaller size of AgNPs than the basal culture condition.

### Characterization of AgNPs

**FIGURE 4:**
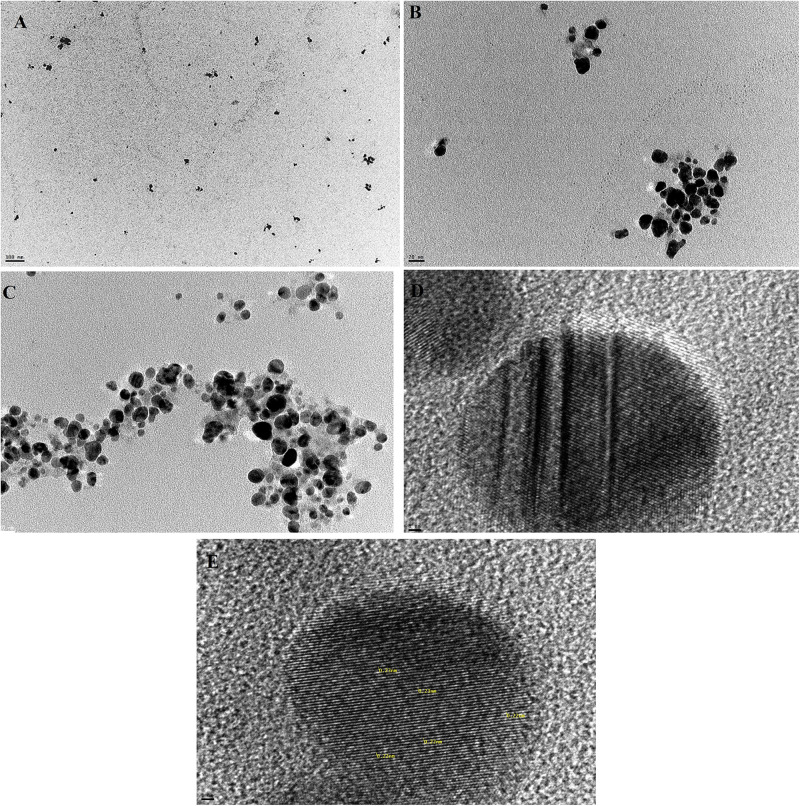
Mycosynthesized AgNPs from *A. terreus* BA6 characterized by HRTEM **(A)** 70,000×, **(B)** 500,000×, **(C)** 500,000× showing the coat around the particles, **(D)** 10,000,000× showing the coat around the particles, and **(E)** 10,000,000× showing the lattice planes inside the crystal.

**FIGURE 5:**
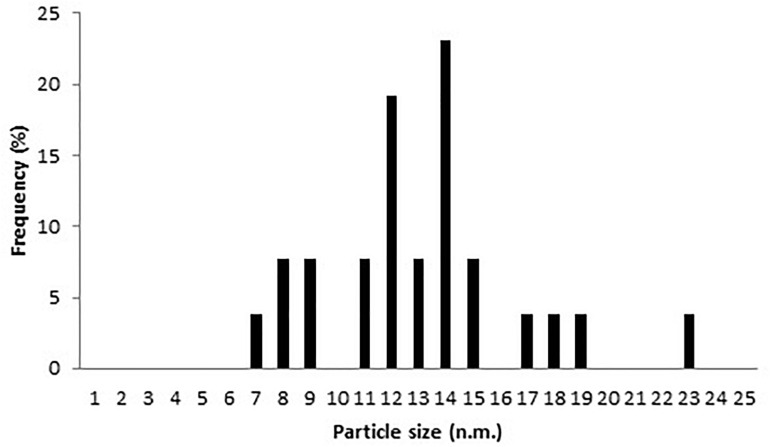
Nanoparticles size distribution of AgNPs synthesized by *A. terreus* BA6.

AgNPs mycosynthesized by *A. terreus* BA6 under near optimal conditions were subjected to HRTEM to determine their structure. The micrograph showed that nanoparticles were spherical in shape, well dispersed and properly separated without any agglomerations (Figure 4). A white layer appeared around many particles which might act like a coat preventing particles agglomeration (Figures 4C,D). In addition, AgNPs showed a crystalline structure with a distance of 0.22–0.23 nm between lattice planes (Figure 4E). Furthermore, the histogram (Figure 5) showed narrow size distribution ranging from 7 to 23 nm and the most frequent size of AgNPs was 14 nm.

**FIGURE 6:**
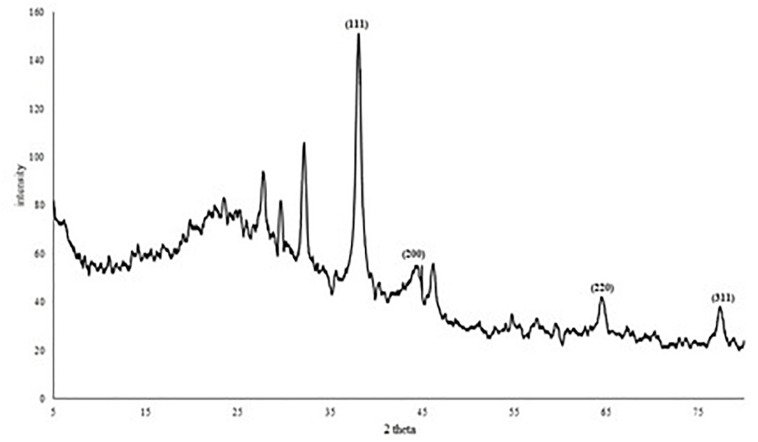
XRD diffractogram of AgNPs mycosynthesized by *A. terreus* BA6.

The XRD pattern (Figure 6) of AgNPs revealed four diffraction peaks at 2θ = 38.06°, 44.39°, 64.4° and 77.29° that are analogous to (111), (200), (220), and (311) lattice plane values, respectively. This is corresponding to face-centered cubic (FCC) structure of metallic silver (International Center for Diffraction Data, ICDD, silver files No. 00-001-1164 and No. 04-0783). In addition, the diffractogram showed another four peaks at 27.74°, 29.63°, 32.155°, and 46.18° which may correspond to reducing and capping organic moieties attached to AgNPs. Based on the calculation of particles size (D) and *d*-spacing (*d*) using Debye-Scherrer formula and Bragg’s law, the average diameter (D) of the synthesized AgNPs was 15.82 ± 1.73 nm with a distance between planes of atoms inside the particle (*d*) of 0.176 ± 0.05 nm (Supplementary Table 4).

**FIGURE 7:**
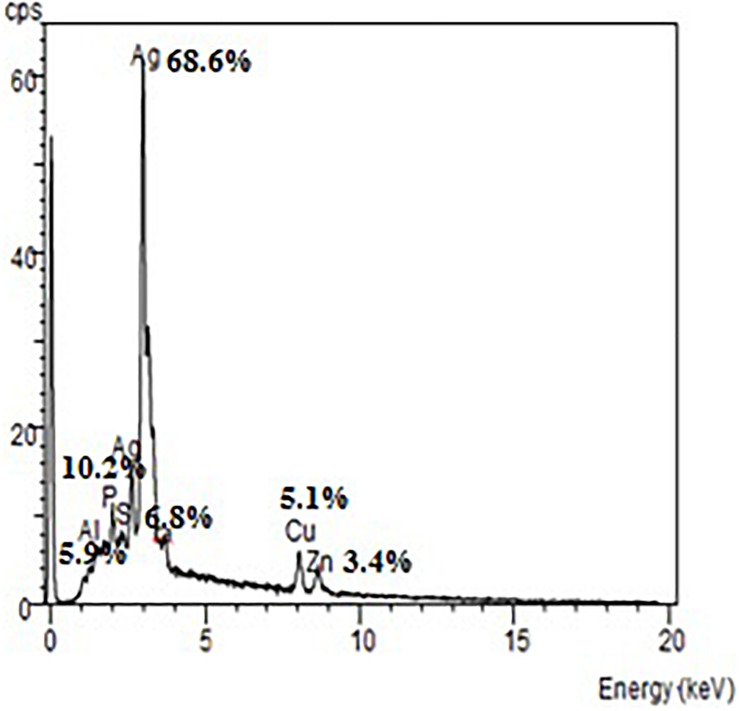
EDX spectrum of *A. terreus* BA6 AgNPs.

**FIGURE 8:**
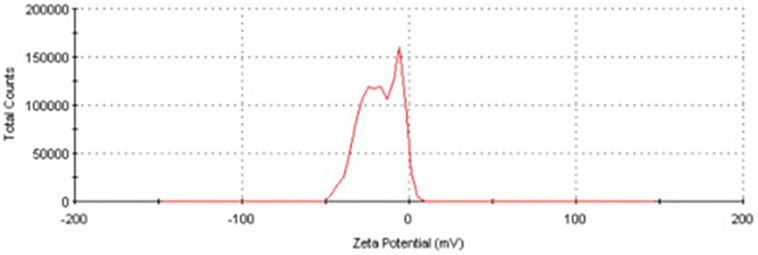
Zeta potential distribution of AgNPs produced by *A. terreus* BA6 under near optimum conditions.

The EDX spectrum (Figure 7) showed a strong signal at 3 keV that was attributed to the SPR of Ag nano-crystals (68.6%) and weaker signals from Al (5.9%), P (10.2%), S (6.8%), Cu (5.1%), and Zn (3.4%) atoms. The copper element of the copper grid onto which the sample was loaded caused the appearance of a peak at 8 keV. The DLS analysis presented in Figure 8 revealed that the average value of surface charge (ξ) was −17.5 mV.

**FIGURE 9:**
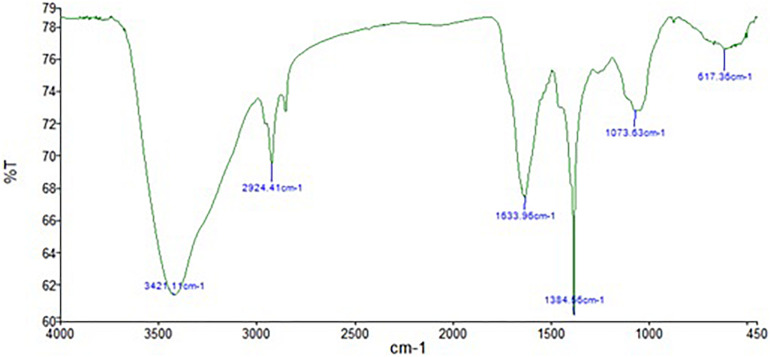
FTIR spectrum of AgNPs obtained from *A. terreus* BA6.

**FIGURE 10:**
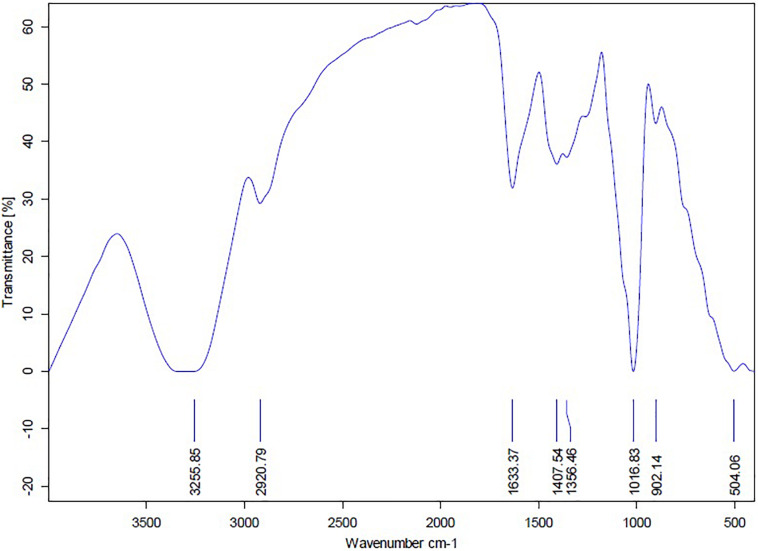
FTIR spectrum of mycelial extract of *A. terreus* BA6.

The FTIR analysis of AgNPs (Figure 9) showed intensive peaks at 3421.1, 2924.41, 1633.96, 1384.56, 1073.63, and 617.36 cm^–1^. Those peaks were, respectively, corresponding to N-H stretching of primary amine of the protein, alkane C-H stretching, the stretching of conjugated alkane C=C, methylene tails of the protein (CH_3_-R), C-N of aliphatic amines of polyphenols, and O-H stretching. The FTIR analysis of the mycelial extract (Figure 10) showed intensive peaks at 3255.85, 2920.79, 1633.37, 1407.54, 1356.46, 1016.83, 902.14, and 504.06 cm^–1^. Those peaks were, respectively, corresponding to O-H stretching of alcohols or phenols, alkane C-H stretching, N-H stretching of amines, C-C aromatic stretching, C-H rocking vibration of alkanes, C=O stretching of carboxylic acid, N-H stretching of amines, and ring C-C-C symmetric bending. On the other hand, stability of AgNPs was determined by measuring the UV absorbance, the results showed particle stability at up to 3 months of synthesis (Supplementary Figure 3).

### Determination of the Bioactivity of AgNPs Mycosynthesized by *A. terreus*

#### Antimicrobial Activity

The MIC, MBC, and MFC values of AgNPs versus standard antibiotics against 12 ATCC standard microorganisms are shown in Supplementary Table 5. *P. aeruginosa*, *Streptococcus faecalis* and *A. niger* showed the highest susceptibility to AgNPs at 0.312 μg/ml MIC. On the other hand, *E. coli* and *C. albicans* showed the highest resistance to AgNPs at 1.25 μg/ml MIC. Furthermore, AgNPs showed remarkable bactericidal (MBC = 0.625–10 μg/ml) and fungicidal (MFC = 10 μg/ml) activities against all tested bacterial and fungal strains. The MIC and MBC/MFC results of AgNPs were comparable to those of the standard antibiotics gentamicin, vancomycin, and fluconazole used for Gram negative bacteria, Gram positive bacteria, and fungi, respectively. The MIC values of gentamicin, vancomycin, and fluconazole were, respectively, 0.019 to 0.312 μg/ml, 0.039 to 0.156 μg/ml, and 0.156 μg/ml. Also, their observed MBC values were, respectively, 0.312 to 5 μg/ml, 0.625 to 5 μg/ml, and 5 μg/ml. Turbidity was observed in the culture media that contained the fungal filtrate solely, indicating the absence of any antimicrobial activity against the indicator microorganisms. No turbidity was observed in the wells that contained only MHB confirming the sterility of media and microtiter plates.

#### Antiviral Activity Against Coxsackie B Virus

MTT assay results revealed that AgNPs produced by *A. terreus* BA6 were not able to inhibit infectivity of Vero cells by Coxsackie B virus (data not shown). The effect of the MNTC of AgNPs (43.75 μg/ml) on Coxsackie B virus infected Vero cells is shown in Supplementary Figure 4.

#### Cytotoxic Effect Against Mcf-7 Cell Line

**FIGURE 11:**
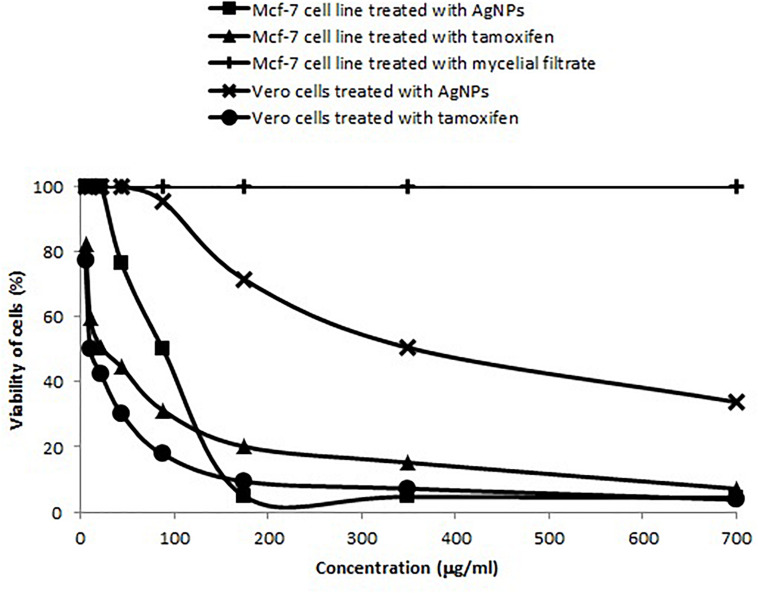
Concentration dependent cytotoxic effects of AgNPs synthesized by *A. terreus* BA6 and tamoxifen on adenocarcinoma human breast cells (Mcf-7).

The IC_50_ of *A. terreus* nano-Ag against Mcf-7 and Vero cell lines were estimated from the data illustrated in Figure 11. Mcf-7 cells were more sensitive to AgNPs showing an IC_50_ of 87.5 μg/ml followed by Vero cells that exhibited an IC_50_ of 350 μg/ml. The increase in the concentration of AgNPs was concomitant with higher cytotoxic activity against Mcf-7 cell line. Furthermore, the MNTC of AgNPs for Mcf-7 and Vero cell lines were, respectively, found to be 21.87 and 43.75 μg/ml, and the calculated SI value for AgNPs against Mcf-7 was 4.0. On the other hand, the IC_50_ of tamoxifen against Mcf-7 and Vero cell lines were 21.87 and 10.39, respectively. The percentages of minimum cell viability of AgNPs and tamoxifen treated Mcf-7 cells were, respectively, 4.43 and 7.2% at 700 μg/ml. The effects of AgNPs, mycelial extract and tamoxifen on Mcf-7 cells are shown in Supplementary Figure 5. The cytotoxic effect of AgNPs against Mcf-7 cells was dose dependent as indicated by the damage observed in Mcf-7 cells. On the other hand, the mycelial filtrate did not show any morphological changes in Mcf-7 cells when compared to the control untreated cells.

## Discussion

Fungal synthesis of AgNPs offers various advantages over their biosynthesis by plants, since scaling-up and optimal culture conditions can be easily achieved to obtain the desired shape and size of AgNPs. Moreover, contrary to the chemical synthesis of AgNPs, the biological approach is non-toxic, inexpensive, and stable. AgNPs have been incorporated in various industrial and medical applications due to the uniqueness of their physio-chemical characteristics (Abdelghany et al., 2017). The first step carried out in the current study was to screen for a competent AgNPs producer fungal isolate. The fungus was isolated from soil and was designated as *A. terreus* strain BA6 based on ITS1, 5.8S, and ITS2 sequences of rRNA gene.

To the best of our knowledge, optimization of *A. terreus* nano-Ag synthesis using DOE for a consistent scaling-up has not been reported so far. Therefore, the Plackett-Burman was adopted to define the most optimal levels of the factors affecting AgNPs synthesis by *A. terreus* (Ghanem et al., 2020). Accordingly, increasing the levels of AgNO_3_ concentration and medium pH was advantageous for the production of smaller AgNPs by *A. terreus* BA6. The positive main effect of reaction pH is in keeping with the results reported by Singh et al. (2014) on *Penicillium fellutanum* and Husseiny et al. (2015) on *Fusarium oxysporum* for extracellular synthesis of AgNPs. The optimum AgNO_3_ concentration depends on the fungal filtrate composition which differs from a fungal strain to another (Balakumaran et al., 2016). In the current study increasing AgNO_3_ concentration up to 4.26 resulted in smaller AgNPs which is in accordance with the results reported by Balakumaran et al. (2016). They reported that increasing AgNO_3_ concentrations from 2 to 4 mM enhanced the extracellular production of AgNPs by *A. terreus*. Our results also revealed that mixing equal volumes of AgNO_3_ (4.26 mM) with fungal extract was found to be better for the synthesis of smaller AgNPs. Our finding is consistent with the data reported by Sarsar et al. (2016) on extracellular synthesis of AgNPs by *A. fumigates* MA. To our knowledge, no previous studies have been reported on the simultaneous effect of inoculum size, filtrate to AgNO_3_ ratio, AgNO_3_, glucose and peptone concentration on the size of AgNPs synthesized by *A. terreus*.

For the characterization purpose of *A. terreus* nano-Ag, we employed several analytical techniques including UV-visible spectroscopy, HRTEM, XRD, EDX, zeta potential, and FTIR (Zhang et al., 2016; Abdelghany et al., 2017). The microscopic images showed a coat around many AgNPs which is in agreement with the data published by Abd El-Aziz et al. (2012) who reported the presence of peptides and amino acids coat covering AgNPs and preventing their agglomeration. The XRD pattern of AgNPs formed by *A. terreus* BA6 approved their crystalline nature and was consistent with other reports of biosynthesized nano-Ag (Abdel-Hadi et al., 2014; Ottoni et al., 2017). The other four peaks appearing in the diffractogram could be due to the existence of reducing and capping organic moieties attached to AgNPs as reported by other researchers (Mehta et al., 2017; Ottoni et al., 2017).

Particle size, shape, and homogeneity of AgNPs are highly influencing their properties and applications (Wanarska and Maliszewska, 2019). The calculations of particle size and *d*-spacing were consistent with the values measured by HRTEM confirming the accuracy of results. It was obvious that AgNPs diameters estimated by DLS were much higher than that estimated by TEM and XRD. This might be due to the presence of hydrated capping agents and by the solvation effects in which the hydrodynamic diameter could be 1.2 higher than the original diameter of the capped particle (Mukherjee et al., 2008).

A strong signal at 3 keV was detected in the EDX spectrum which was attributed to the SPR of Ag nano-crystals confirming their crystalline nature (Ottoni et al., 2017). The weaker signals of Al, P, S, Cu, and Zn atoms that were detected may be due to the presence of molecular elements or mycelial molecules attached to AgNPs (Palani Velan et al., 2014). On the other hand, the negative value of particles surface charge explains well the dispersity, long term stability, and high colloidal nature of AgNPs due to particles repulsion (Mukherjee et al., 2014; Jyoti et al., 2016). This surface charge could prevent agglomeration and thus ensures particles stabilization (Salem et al., 2011). This is in agreement with the results reported by Balakumaran et al. (2016) who demonstrated negative zeta potential estimation of mycosynthesized AgNPs by *A. terreus*. The FTIR spectrum of AgNPs indicated the presence of alcoholic and phenolic compounds in addition to proteins that could be related to biomolecules involved in the reduction of silver ions and particles stabilization (Ebrahiminezhad et al., 2017; Rani et al., 2017).

MIC, MBC, and MFC of AgNPs reported in the current study were comparable to standard antibiotics and were lower than other reports (Wady et al., 2014; Balakumaran et al., 2016; Erjaee et al., 2017; Patra and Baek, 2017). The implication of this finding confirms the antimicrobial potency of AgNPs prepared using *A. terreus* BA6. Several mechanisms were proposed for the antibacterial activity of AgNPs including enzymes degradation, cellular proteins inactivation or DNA damaging (Wang L. et al., 2017). Bacterial cell lysis could be caused by attachment of AgNPs to the bacterial surface, disrupting its permeability and then penetrating inside the cell causing further damages by interaction with sulfur and phosphorus containing compounds like DNA (Patra and Baek, 2017). Conversely, *A. terreus* nano-Ag did not show any antiviral activity against Coxsackie B virus.

On the other hand, the synthesized AgNPs exhibited an auspicious antitumor activity against Mcf-7 cell line. Our results clearly demonstrated that the IC_50_ of AgNPs mycosynthesized by *A. terreus* against Mcf-7 cell line was relatively close to other studies. Previous studies by El-Sonbaty (2013) and Sherif et al. (2015) have shown that the IC_50_ of AgNPs synthesized by *F. oxysporum* and *Agaricus bisporus* on Mcf-7 were 50 and 121.23 μg/ml, respectively. The SI value for AgNPs against Mcf-7 was greater than 2.0 which indicates a high degree of selective toxicity against Mcf-7 cells (Artun et al., 2016). In this context, the findings obtained in the present work may open several avenues of further studies on the antitumor activity of nano-Ag from *A. terreus* BA6 against other cell lines. Tamoxifen is the drug of choice for early-stage and metastatic estrogen receptor-positive breast cancer. Interestingly, our results demonstrate that AgNPs of *A. terreus* are more toxic to Mcf-7 cells than normal Vero cells, whereas tamoxifen is more toxic to Vero cells than Mcf-7 cells. This may be explained on the basis of ROS induction by AgNPs; at cellular levels, higher concentrations of ROS cause oxidative stress to proteins and nuclear materials which induces apoptosis in a variety of human cancers (Han et al., 2014; Wang T.-Y. et al., 2017). Moreover, tumor cells depend on mitochondrial ATP production to manage an extensive metabolic rewiring and to survive in severe nutritional conditions. Therefore, suppression of mitochondria oxidative phosphorylation by AgNPs can also induce toxicity to cancer cells (Fan et al., 2014).

## Conclusion

The current study used *A. terreus* as an effective biofactory for AgNPs biosynthesis. Various techniques were used to characterize AgNPs and to accurately define their physical and morphological properties. Moreover, optimization of AgNPs synthesis by DOE resulted in 26.7% smaller particles than the basal culture condition. The mycosynthesized AgNPs possess significant antimicrobial activity against various reference pathogens in addition to their cytotoxic effect against Mcf-7 cell line. In future, it would be important to study the cytotoxicity of *A. terreus* nano-silver against other malignant cell lines as well as its antiviral activity against putative and emergent viruses.

## Data Availability Statement

The datasets presented in this study can be found in online repositories. The names of the repository/repositories and accession number(s) can be found in the article/Supplementary Material.

## Author Contributions

SS, HG, and WL designed the study and supervised all of the experimental works. BA carried out the experiments. SS, HG, WL, and BA analyzed the data. WL wrote the manuscript. All authors contributed to the article and approved the submitted version.

## Conflict of Interest

The authors declare that the research was conducted in the absence of any commercial or financial relationships that could be construed as a potential conflict of interest.

## Footnotes

1 https://www.ncbi.nlm.nih.gov/nuccore/MG725681
