# Supplementary material for: Biosynthesis of Silver Nanoparticles by Aspergillus terreus: Characterization, Optimization, and Biological Activities
Source: Front Bioeng Biotechnol. 2021 Apr 15;9:633468. doi: 10.3389/fbioe.2021.633468 (PMC8081910; doi:10.3389/fbioe.2021.633468)
Supplement: Supplementary file 1 [file Data_Sheet_1.pdf]

Supplementary Material

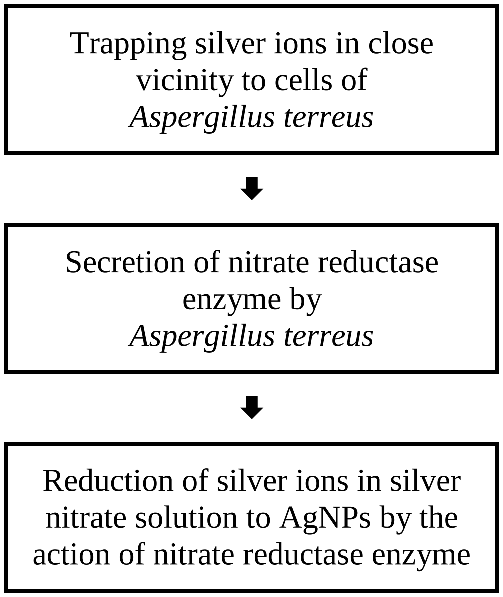

Supplementary FIGURE 1 Schematic diagram of AgNPs synthesis mechanism.

Supplementary TABLE 1 Matrix of the Plackett-Burman experimental design.

Trial	Independent variable							Average diameter of AgNPs (nm)
	Medium pH	Mycelial filtrate to silver nitrate ratio	Reaction pH	Inoculum size (spores/ml)	Dextrose (g/l)	Peptone (g/l)	Silver nitrate (mM)	
**1**	5	0.5	6.25	10^7	27.5	12.5	0.55	Negative
**2**	9	0.5	6.25	10^5	12.5	12.5	5.50	1378
**3**	5	1.0	6.25	10^5	27.5	7.5	5.50	100.4
**4**	9	1.0	6.25	10^7	12.5	7.5	0.55	Negative
**5**	5	0.5	8.75	10^7	12.5	7.5	5.50	729.3
**6**	9	0.5	8.75	10^5	27.5	7.5	0.55	180.9
**7**	5	1.0	8.75	10^5	12.5	12.5	0.55	Negative
**8**	9	1.0	8.75	10^7	27.5	12.5	5.50	1298
**9**	7	0.75	7.50	10^6	20.0	10.0	1.00	72.14
**10**	7	0.75	7.50	10^6	20.0	10.0	1.00	68.28
**11**	7	0.75	7.50	10^6	20.0	10.0	1.00	68.28

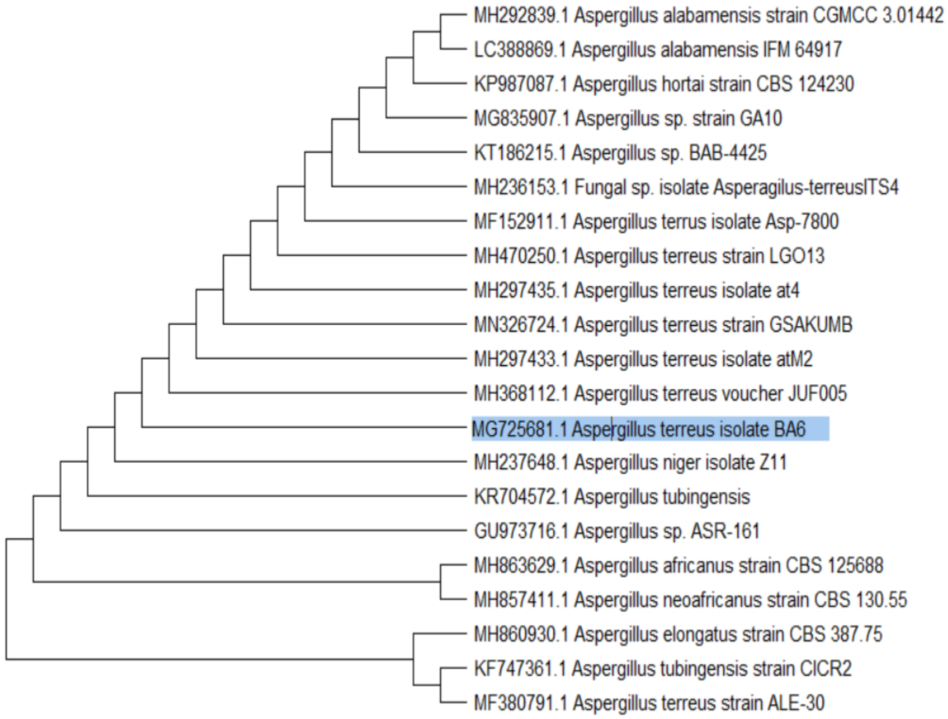

Supplementary FIGURE 2 The phylogenetic relationship between *A. terreus* BA6 and related fungal strains retrieved from NCBI GenBank.

Supplementary TABLE 2 Statistical analysis of the Plackett-Burman experimental results.

Variable	ANOVA				Main effect and significance				
	SS	df	MS	F	Coefficient	Main effect	*t*-test	*p*-value	Significance level (%)
**Intercept**	--	--	--	--	458.111	458.11	143.675	0.000001	99.9
**Medium pH**	513 692	1	513 692	5054.07	253.400	506.80	71.092	0.000006	99.9
**Mycelial filtrate to $AgNO_3$**	98 968	1	98 968	973.72	-111.225	-222.45	-31.205	0.000072	99.9
**Reaction pH**	66 576	1	66 576	655.02	91.225	182.45	25.593	0.000131	99.9
**Inoculation size (spores/ml)**	21 474	1	21 474	211.27	48.221	96.44	14.535	0.000706	99.9
**Dextrose (g/l)**	34 848	1	34 848	342.86	-66	-132.00	-18.517	0.000344	99.9
**Peptone (g/l)**	346 695	1	346 695	3411.03	208.175	416.35	58.517	0.000011	99.9
**$AgNO_3$ (mM)**	1 612 204	1	1 612 204	15 862.01	417.821	835.64	125.944	0.000001	99.9
**Error**	305	3	102						
**Total**	2 793 503	10							

Supplementary TABLE 3 Settings of the validation experiment.

Factor	Culture condition		
	Near optimum	Basal	Anti-optimized
**Medium pH**	5.00	7.00	9.00
**Mycelial filtrate: $AgNO_3$**	1.00	0.75	0.50
**Reaction pH**	6.25	7.50	8.75
**Inoculation size (spores/ml)**	10^5	10^6	10^7
**Dextrose (g/l)**	27.50	20.00	12.50
**Peptone (g/l)**	8.75	10.00	12.50
**Ag NO_3 (mM)**	4.26	1.00	0.55

Supplementary TABLE 4 Particles size and *d*-spacing of AgNPs mycosynthesized by *A. terreus* BA6.

**Peak khl**	**Ɵ**	**β**	**λ**	**D**	***d***
111	19.0333	0.01093	1.54606	13.46	0.2370
200	22.195	0.00942	1.54606	15.95	0.2040
220	32.2	0.01011	1.54606	16.26	0.1450
311	38.645	0.01011	1.54606	17.62	0.1200

[b]Ɵ, the diffraction angle; β, full width at half maximum in radians; λ, wavelength of X-ray (nm); D, particles diameter (nm); and *d*, the distance between planes of atoms inside the crystal (nm).

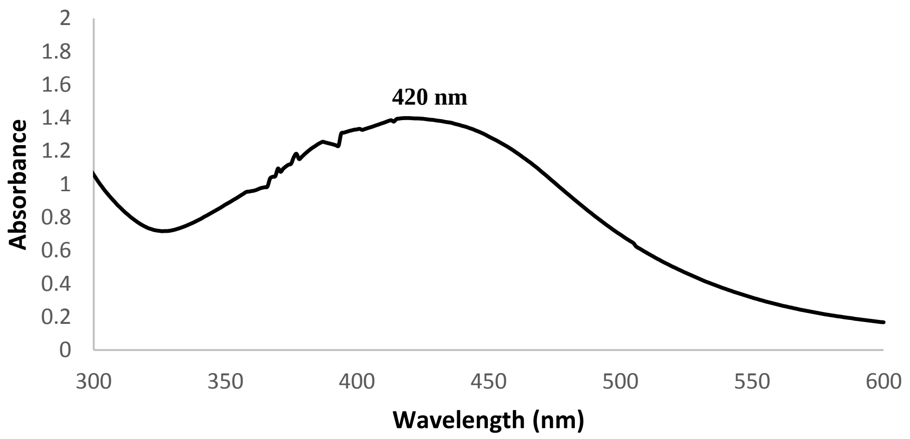

Supplementary FIGURE 3 UV-Visible absorption spectrum of *A. terreus* BA6 AgNPs after 3 months of synthesis.

Supplementary TABLE 5 MIC, MBC and MFC of AgNPs synthesized by *A. terreus* BA6 against ATCC standard microorganisms.

Pathogen	MIC (µg/ml)		MBC/MFC (µg/ml)	
	AgNPs	Control antibiotic*	AgNPs	Control antibiotic*
Escherichia coli ATCC 8739	1.250	0.312	5.000	1.250
Salmonella typhimurium ATCC 14028	0.625	0.312	5.000	5.000
Pseudomonas aeruginosa ATCC 9027	0.312	0.039	0.625	0.312
Aeromonas hydrophila ATCC 35654	0.625	0.019	2.500	1.250
Bacillus subtilis ATCC 6633	0.625	0.039	5.000	2.500
Staphylococcus aureus ATCC 6538	0.625	0.156	1.250	0.625
Staphylococcus epidermidis ATCC 12228	0.625	0.156	10.000	5.000
Streptococcus faecalis ATCC 10541	0.312	0.156	5.000	2.500
Methicillin resistant *Staphylococcus aureus* (MRSA) ATCC 43300	0.625	0.156	2.500	1.250
Listeria monocytogenes ATCC 19111	0.625	0.039	5.000	2.500
Candida albicans ATCC 10231	1.250	0.156	10.000	5.000
Aspergillus niger ATCC 16404	0.312	0.156	10.000	5.000

* Gentamicin, vancomycin, and fluconazole are the control antibiotics used for Gram negative bacteria, Gram positive bacteria and fungi, respectively.

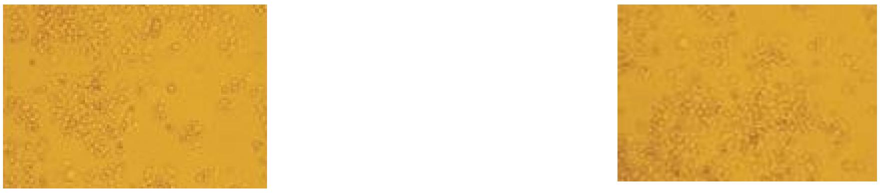

A **B**

Supplementary FIGURE 4 (A) Effect of Coxsackie B virus on Vero cells, (B) Coxsackie B virus infected Vero cells treated with the maximum non-toxic concentration of AgNPs (43.75 μg/ml).

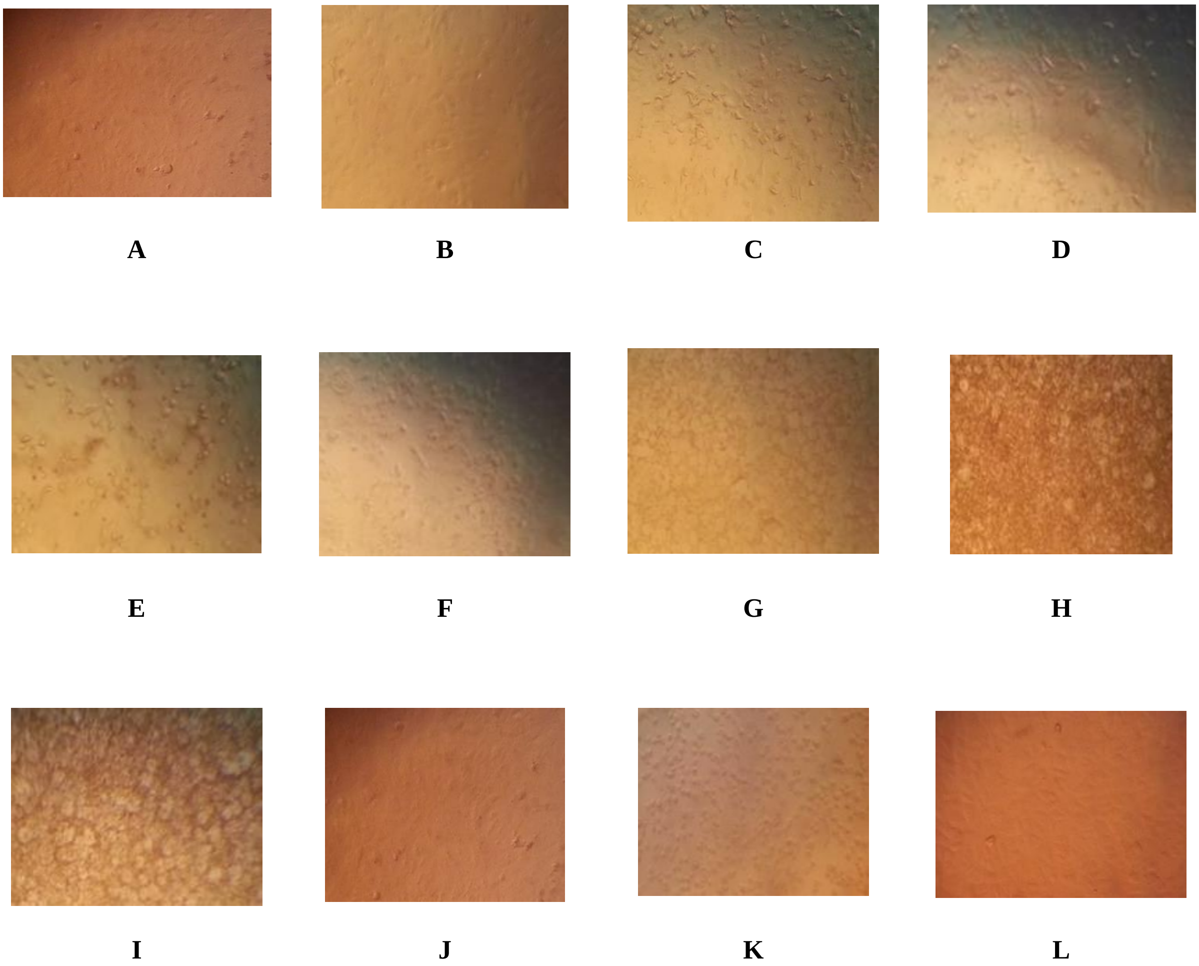

Supplementary FIGURE 5 (A) Control Mcf-7 cells, (B-I) Mcf-7 cells treated with AgNPs 5.46, 10.93, 21.87, 43.75, 87.5, 175, 350, and 700 µg/ml, respectively, (J) Mcf-7 cells treated with mycelial filtrate, (K) Mcf-7 cells treated with 700 µg/ml tamoxifen, (L) Vero cells treated with the maximum non-toxic concentration of AgNPs (43.75 µg/ml).
